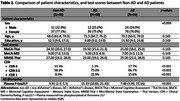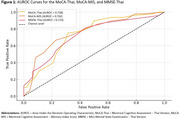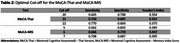# Performance of Cognitive Screening Tests for Alzheimer's Disease Pathology Defined by Plasma *p*‐tau217: A Prospective Cohort Study in Early Dementia Patients at King Chulalongkorn Memorial Hospital, Thailand

**DOI:** 10.1002/alz70857_107090

**Published:** 2025-12-26

**Authors:** Yuthachai Sarutikriangkri, Thanakit Pongpitakmetha, Akarin Hiransuthikul, Tara Rak‐areekul, Adipa Chongsuksantikul, Prawit Oangkhana, Watayuth Luechaipanit, Thanaporn Haethaisong, Yuttachai Likitjaroen, Poosanu Thanapornsangsuth

**Affiliations:** ^1^ Memory Clinic, King Chulalongkorn Memorial Hospital, The Thai Red Cross Society, Bangkok, Thailand; ^2^ Neurocognitive Unit, Division of Neurology, Faculty of Medicine, Chulalongkorn University, Bangkok, Thailand; ^3^ Department of Pharmacology, Faculty of Medicine, Chulalongkorn University, Bangkok, Thailand; ^4^ Division of Neurology, Department of Medicine, Faculty of Medicine, Chulalongkorn University, Bangkok, Thailand; ^5^ Chula Neuroscience Center, King Chulalongkorn Memorial Hospital, The Thai Red Cross Society, Bangkok, Thailand; ^6^ Department of Preventive and Social Medicine, Faculty of Medicine, Chulalongkorn University, Bangkok, Thailand; ^7^ Thai Red Cross Emerging Infectious Diseases Health Science Centre, World Health Organization Collaborating Centre for Research and Training on Viral Zoonoses, King Chulalongkorn Memorial Hospital, The Thai Red Cross Society, Bangkok, Thailand

## Abstract

**Background:**

Plasma *p*‐tau217 is emerging as a biomarker for Alzheimer's disease (AD) diagnosis, offering a more accessible alternative to CSF and amyloid‐PET. The Montreal Cognitive Assessment – Thai Version (MoCA‐Thai) and Mini‐Mental State Examination – Thai Version (MMSE‐Thai) are widely used to detect cognitive impairment in clinical settings, but their optimal cut‐off scores for identifying AD pathology, particularly with plasma *p*‐tau217, remain unclear, especially in lower‐ and middle‐income countries (LMICs). This study evaluates the performance of MoCA‐Thai, MoCA–Memory Index Score (MoCA‐MIS), and MMSE‐Thai for AD diagnosis using plasma *p*‐tau217.

**Methods:**

We recruited patients with early‐stage dementia (CDR ≤ 1) from the INDE cohort at King Chulalongkorn Memorial Hospital, Thailand (NCT06375213). AD pathology was determined using an internally validated plasma *p*‐tau217 cutoff (>7.46 pg/mL). Cognitive assessments and Clinical Dementia Rating (CDR) scoring were conducted by trained clinical psychologists. Receiver operating characteristic (ROC) analysis and Youden's index were used to determine optimal cut‐off scores.

**Results:**

There were no significant differences in age, sex, or education level between AD and non‐AD groups (Table 1). However, AD patients had significantly lower scores on MoCA‐Thai, MoCA‐MIS, and MMSE‐Thai (*p* < 0.001). ROC analysis showed that MoCA‐MIS (AUROC = 0.762) had the highest discriminative ability, followed by MoCA‐Thai (AUROC = 0.738) and MMSE‐Thai (AUROC = 0.725) (Figure 1). Optimal cut‐off scores were determined as ≤21 for MoCA‐Thai (Sensitivity = 75%, Specificity = 69%) and ≤6 for MoCA‐MIS (Sensitivity = 67%, Specificity = 77%) (Table 2).

**Conclusion:**

In our cohort, a MoCA‐Thai cut‐off of ≤21 and a MoCA‐MIS cut‐off of ≤6 provided the best optimal sensitivity and specificity for detecting AD pathology. These findings support the integration of cognitive screening tests with plasma biomarkers to enhance early AD detection in clinical settings in Thailand, where access to advanced diagnostics is limited.